# The impact of associated tenotomies on the outcome of incomplete phalangeal osteotomies for lesser toe deformities

**DOI:** 10.1186/s13018-019-1353-0

**Published:** 2019-09-11

**Authors:** Eduardo Nieto-García, Javier Ferrer-Torregrosa, Leonor Ramírez-Andrés, Elena Nieto-González, Alfonso Martinez-Nova, Carlos Barrios

**Affiliations:** 1 Doctorate School, Valencia Catholic University San Vicente Martir, Valencia, Spain; 2 School of Physiotherapy and Podiatry, Valencia Catholic University, Ramiro Maeztu 14, 46900 Torrent, Valencia, Spain; 3 Podiatric School, University of Extremadura, Plasencia, Cáceres, Spain; 4 Institute for Research on Musculoskeletal Disorders, Valencia Catholic University, Valencia, Spain

**Keywords:** Lesser toe deformities, Percutaneous osteotomies, Tenotomy, Forefoot surgery, Minimally invasive surgery, Complications

## Abstract

**Background:**

Partial or incomplete osteotomy (IO) of the phalanx is recently described in the literature. However, the clinical outcome and the rate of complications when applied to lesser toe deformities (LTD) have been never addressed. This study aims to find out if the association of tenotomies to incomplete or partial phalanx osteotomies has a significant impact on the clinical outcomes, the occurrence of complications, and the recovery time after surgery.

**Methods:**

A retrospective review of two cohorts of cases operated in our institution for hallux abductus valgus (HAV) and associated LTD from 2008 to 2014 was carried out. The surgical correction of both HAV and the associated LTD was always performed by minimally invasive techniques. The study included a total of 223 patients (723 IO in 556 toes). In 129 cases, the IO for LTD correction was performed without tenotomies, and in 94, the procedure was combined with flexor and/or extensor tenotomies. Patients were assessed with the American Orthopaedic Foot and Ankle Society (AOFAS) questionnaire before surgery and at 6- and 12-month follow-up.

**Results:**

The mean preoperative AOFAS score before surgery was similar in both cohorts. At 12-month follow-up, the cohort without tenotomies showed better recovery (95.7 ± 2.8 versus 92.5 ± 6.8; *p* < 0.01). AOFAS scores decreased as the number of associated LTD increased (*r* = − 0.814; *p* < 0.001). Cases operated on by PO + tenotomy showed a high rate of complications such as delayed union of the osteotomy (*p* < 0.01), hypertrophic callus (*p* < 0.01), phalangeal fracture at the osteotomy site (*p* < 0.01), and lack of correction (*p* < 0.05). The overall occurrence of adverse events was 38.6% in cases operated by PO + tenotomy and 13.9% in cases receiving PO alone (*p* < 0.0001). Cases operated on without tenotomy showed a shorter time to complete recovery for daily life activities (37.4 ± 2.3 versus 43.0 ± 1.7 days; *p* < 0.01).

**Conclusion:**

The performance of associated tenotomies to incomplete phalanx osteotomies provides worse clinical outcomes, higher complication rates, and longer recovery time as compared to similar forefoot surgeries without tenotomies.

**Trial registration:**

The study was based on retrospectively registered data starting on May 24, 2008.

## Introduction

Lesser toe deformities (LTD) are highly frequent in the general aging population and may be associated with significant morbidity [1, 2]. These deformities occur gradually, often affect multiple toes, and are regularly associated to hallux abductus valgus (HAV) [3]. The progression of the deformity is commonly related to an imbalance between the forces of the extensor and flexor tendons about the proximal or distal interphalangeal joints [4]. Most of the LTD evolve despite podiatric care and lastly require surgical treatment. A Swedish experience based on extensive data registries suggests that almost a quarter of patients undergoing forefoot surgery had also lesser toe procedures performed [5]. Arthroplasty and arthrodesis are still widely used techniques despite their functional squeals [6–10]. The impact of associated surgeries for correction of LTD in terms of complication occurrence and final recovery after HAV is still a matter of controversy.

Minimally invasive surgery (MIS) involves bony or soft tissue procedures performed through a small incision, without direct visualization on the anatomical structures [11].

MIS techniques are attractive procedures because of their advantages: reduced soft tissue damage, shorter length of surgery and hospital stay, lower post-operative pain, and reduced infection risk. The results seem similar to those obtained using the various open techniques, although the wide range of clinical conditions makes comparisons difficult [11, 12].

One of the MIS procedures for LTD is the partial or incomplete osteotomy (IO) of the phalanges which consists of a unicortical osteotomy retaining an intact portion of the phalanx that may act as a fulcrum allowing the closure of the osteotomy. The first described IO was applied on the proximal phalanx of the first finger (modified Akin technique) [13, 14]. Based on their behavior, the design and execution were transferred to the rest of the phalanges of the minor toes, second to fifth. Multiple IOs can be performed in the same finger with different designs since each digital deformity is different [15, 16].

The goal of IO is to obtain a digital realignment that permits to recover the balance between the extrinsic and intrinsic musculature without needing to perform surgical procedures on tendon structures. In this way, the interphalangeal joints could reestablish their physiological functionality. IOs are techniques especially indicated for reducible or semi-rigid deformities of the fingers [17].

The surgical technique of IO has been well described in the literature [16, 17]. However, the clinical outcome and the rate of complications of IO applied to LTD have never been addressed. This study aims first to analyze the clinical impact of the associated surgical procedures performed at the lesser toes at the time of HAV correction. Second and more specifically, this study evaluates the clinical efficacy and safety of IO procedures for correction of second to fifth digital deformities, focusing the more frequent complications. Third, this study analyzed if the association of tenotomies to IO techniques to correct LTD has a significant impact on the clinical outcomes, the occurrence of complications, and the recovery time after surgery.

## Methods

### Participants

From 2008 to 2014, a total of 457 patients were operated on in our institution for HAV and associated LTD. Out of these, 223 patients were selected for the final study according to the following criteria related to the type of LTD: (i) reducible deformity of the proximal interphalangeal joint in any spatial plane, that is reducible hammer and claw toe; and (ii) soft tissue involvement with extensor and flexor tendon imbalance. Cases with non-reducible deformity of the proximal interphalangeal joint (rigid hammer or claw toes) and those with isolate rigid deformity of the distal interphalangeal joint (mallet toes) were excluded. Patients with previous surgeries of the lesser toes were also excluded.

The mean age of the final 223 patients with reducible hammer or claw toes was 59 years, with a predominance of women (*n* = 212; 95.1%). There were 16 (7.2%) diabetic patients and 6 (2.7%) with rheumatoid arthritis. The right foot was treated in 52.9% of cases.

The surgical procedure for correction of both HAV and the associated LTD was always performed by MIS techniques. Out of the 223 cases, 129 (57.8%) underwent correction of the LTD by phalangeal IO without additional tenotomies, and in 94 (42.2%), the procedure was combined with flexor and/or extensor tenotomies.

Fifteen days prior to surgery, all patients were interviewed in order to perform the preoperative tests and complete the American Orthopaedic Foot and Ankle Society (AOFAS) scale. For the purpose of this study, the lesser metatarsophalangeal-interphalangeal subscale was analyzed [18].

### Surgical procedure

HAV correction was executed in all cases by performing Reverdin and Akin osteotomies following similar criteria of Biz et al. [12]. All procedures were performed under regional anesthesia in an office-based operation theater with fluoroscopic guidance.

**Fig. 1:**
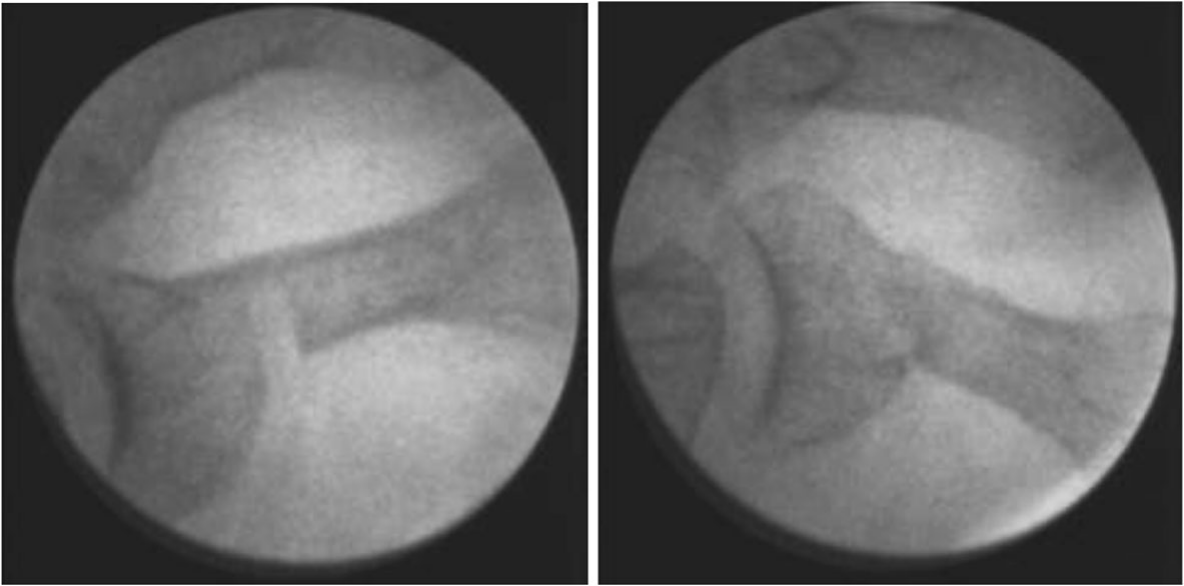
Incomplete phalangeal osteotomy before and after closure

**Fig. 2:**
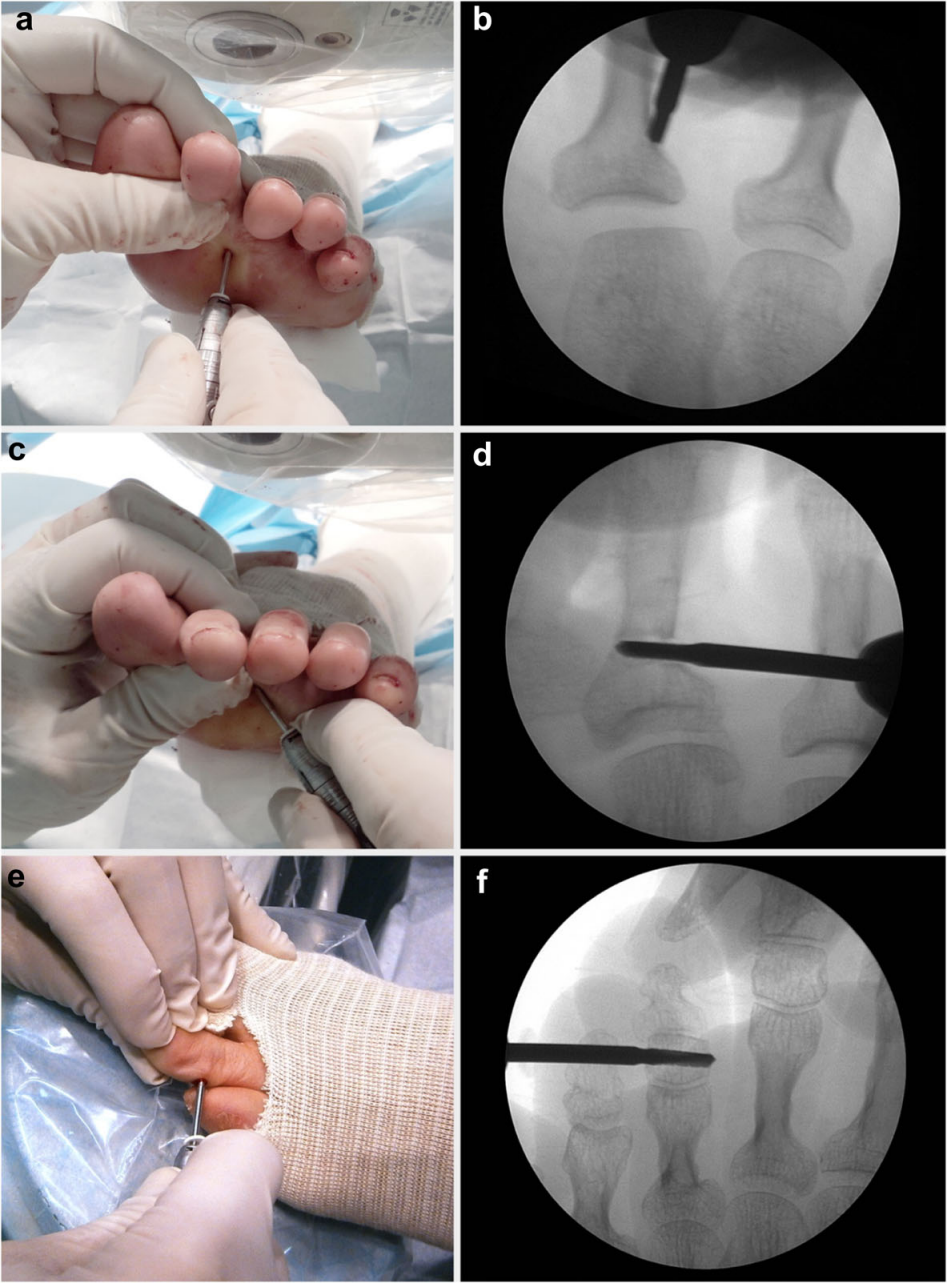
Surgical planification of the incomplete phalangeal osteotomies. **a** Plantar approach of the proximal phalanx of the second toe. **b** Fluoroscopic view of the site of incomplete osteotomy. **c** Final stage of the incomplete osteotomy and **d** the fluoroscopic view. **e** Incomplete osteotomy at the second phalanx of the third lesser toe and **f** the fluoroscopic view

As for the surgical treatment of LTD, the preservation of the cortical continuity at selected point is the differentiating element of IOs as compared to other types of phalangeal osteotomies. The cortical wall acts as a natural fixing device, avoiding the displacement of the fragments and preventing the segment from shortening (Fig. 1). Two particularly relevant features at the time of execution of the phalangeal osteotomy should be considered in this study. The first is that the bone corrections were always performed starting from proximal to distal phalanges (Fig. 2). The second is that once the skeletal segments were realigned, an intra-surgical assessment of the reducibility of the deformity and the equilibrium achieved between the agonist and antagonist muscle groups responsible for the digital movement was assessed. In some cases, the irreducibility of the deformity required to act on the soft tissue structures, that is, on the extensor and/or the flexor digital tendons. The incomplete reducibility of the toe deformity after IO was the criteria to add tenotomies in this series of cases.

### Postoperative protocol

Immediately after surgery, all patients were allowed to full weight-bearing, due to the design of the osteotomies, and the external fixing quality provided by the bandages. Patients were instructed to wear a post-surgical shoe with a rigid sole. Curing of the wounds was performed on a weekly basis. At each postoperative visit, a fluoroscopic check-up was completed to objectify the closure of the osteotomy, the maintenance and stability the phalangeal osteotomies, and adequacy of the bone segment position.

Between 30 and 40 days after surgery, when the formation of fibrous callus was verified in the performed osteotomies and the clinical evolution was satisfactory, the patient was allowed to perform his or her daily activities. Follow-up outpatient visits were carried out 6 and 12 months after surgery for clinical and radiological evolution control, recording again the AOFAS scale. All the complications found such as displacement, non-union, delayed consolidation, hypertrophic callus, and no correction were registered.

### Statistical analysis

This was a retrospective study, and hence, pre-study sample size calculation was not performed. Descriptive statistical tests were first analyzed. Student *t* test, ANOVA, Mann-Whitney test, *χ*^2^ test, and Fisher exact test were used in the statistical analyses of the variables depending on the nature of the variables and their intention for analysis. All statistical tests were performed using SPSS version 21 (SPSS, Inc., IBM, Chicago, IL), and *p* ≤ .05 was considered significant.

## Results

The demographic and clinicopathological characteristics of the two cohorts are summarized in Table 1. There were no differences among the two cohorts in age, sex distribution, laterality, and associated diseases. Of the 223 feet, 556 toes were operated (IO group, *n* lesser toes = 338; IO + tenotomy, *n* lesser toes = 218) with a total of 723 osteotomies (IO group, *n* = 436; IO + tenotomy, *n* = 287).

**Table 1:** Demographic and clinicopathological characteristics of patients

Characteristics	HAV + IO on LT (*n* = 129)	HAV + IO + tenotomy on LT (*n* = 94)	Significance
Age (mean ± SD)	59.6 ± 11.5	59.5 ± 11.3	0.358*
Sex, *n* (%)			
Male	5 (3.9%)	6 (6.4%)	0.533**
Female	124 (96.1%)	88 (93.6%)	
Laterality, *n* (%)			
Right	67 (51.9%)	51 (54.3%)	0.786**
Left	62 (48.1%)	43 (45.7%)	
Associated disease			
Diabetes	6 (4.6%)	10 (10.6%)	0.115**
Rheumatoid arthritis	2 (1.5%)	4 (4.2%)	0.242**

*HAV* hallux abductus valgus, *IO* incomplete osteotomy, *LT* lesser toe

*Unpaired *t* test

**Chi-square test

**Fig. 3:**
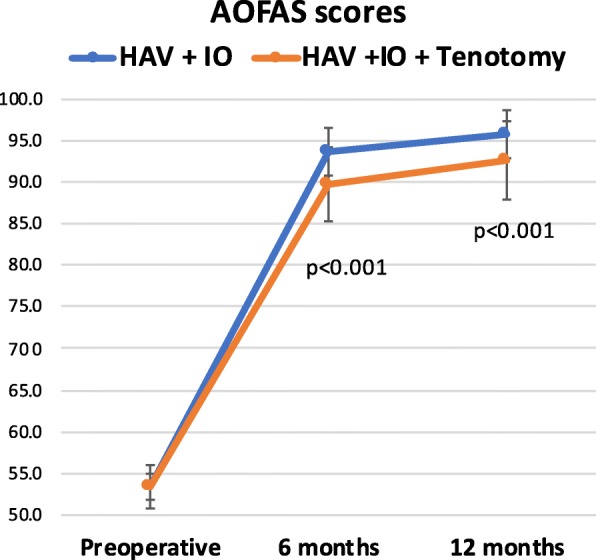
AOFAS scores of the two cohorts during the different follow-up periods

The mean preoperative AOFAS score before surgery was similar in both cohorts of patients with LTD (53.5 ± 10.9 for IO without tenotomy versus 53.4 ± 8.5 in cases with associated tenotomy). Six months after surgery, cases operated with IO alone showed better AOFAS scores than those patients receiving associated tenotomy (93.6 ± 3.4 and 89.7 ± 7.8 respectively; *p* < 0.001) (Fig. 3). Twelve months after surgery, the cohort without tenotomies showed also better recovery according to AOFAS scores (95.7 ± 2.8 for IO without tenotomy versus 92.5 ± 6.8, *p* = 0.001). At 6- and 12-month follow-up, there were no statistically significant differences between the cohorts of isolated HAV and those operated on for LTD with IO without tenotomies (94.5 ± 4.7and 95.7 ± 2.8 respectively).

**Fig. 4:**
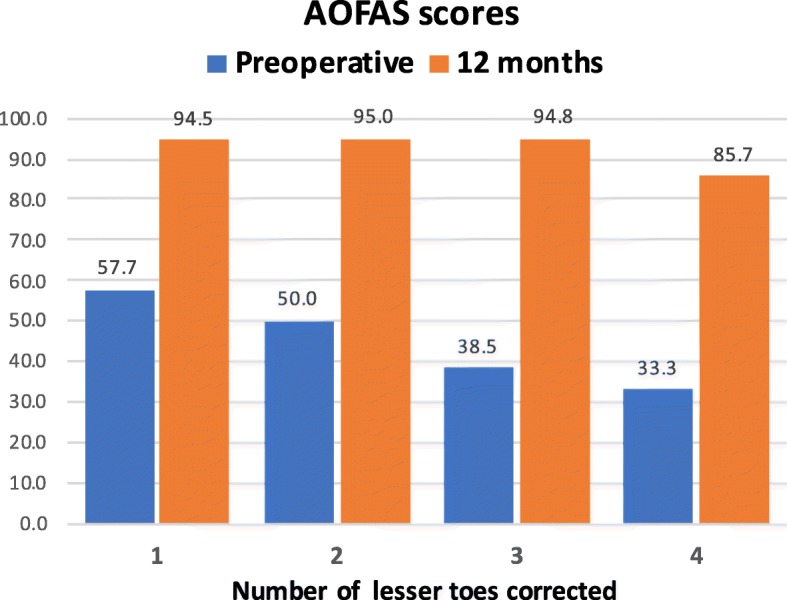
Preoperative and 12-month postoperative AOFAS mean scores discriminating the number of lesser toes operated on

**Fig. 5:**
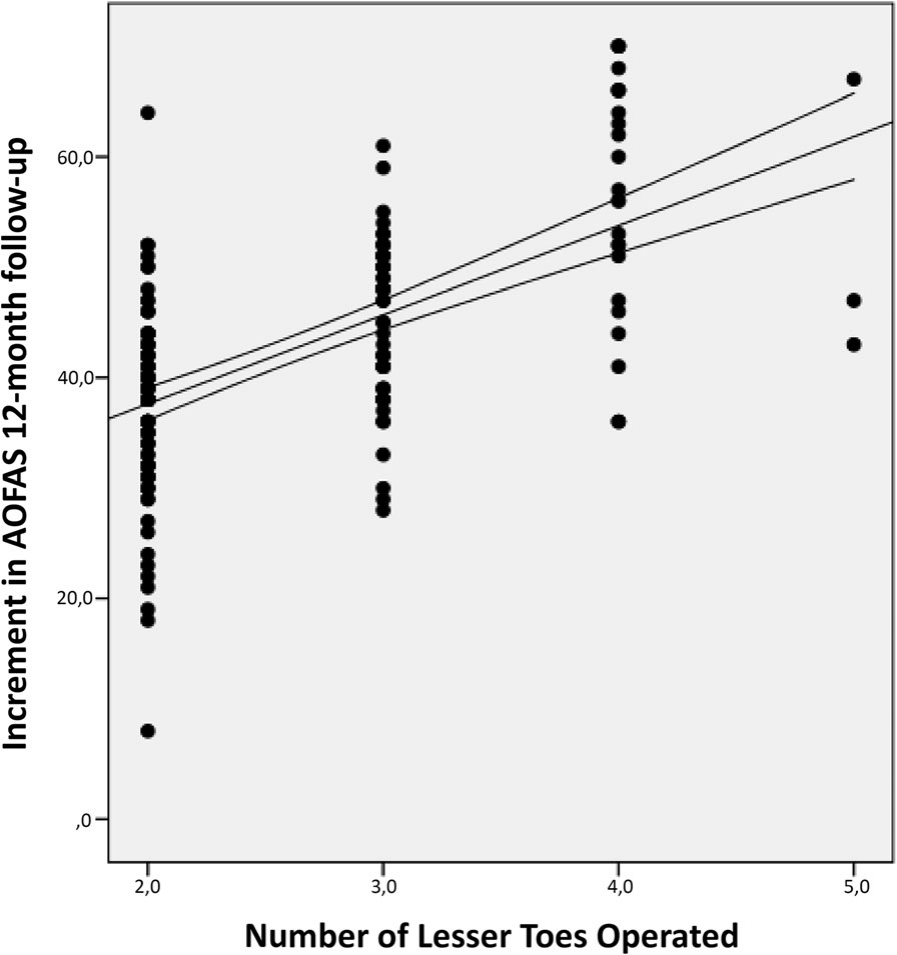
Increment in AOFAS scores from preoperative period to 12-month follow-up in relation to the number of lesser toes operated. The lines indicate the tendency of the mean

Figure 4 shows the preoperative and the 12-month postoperative AOFAS mean scores discriminating the number of lesser toes operated on. The preoperative impairment of the AOFAS scores was dependent of the number of toes requiring surgical correction. The higher scores (less clinical impairment) were recorded in cases operated only of one lesser toe. AOFAS scores decreased as the number of associated LTD increased (*r* = − 0.814; *p* < 0.001) (Fig. 5). Except for the cases requiring correction of all five toes, the recovery was independent of the number of toes operated in association with HAV surgery.

The number of complications related to surgery and the percentage of occurrence regarding the number of operated feet and the number of procedures is summarized in Table 2. Complications of the LTD procedures were accounted separately from those occurring on the first day. When the occurrence of adverse events was considered in relation to the number of performed procedures, the complication rate of IO + tenotomy resulted in 38.6% (111/287), while that of IO alone was 13.9% (61/436), having statistical differences that are highly significant (*p* < 0.0001). Taking all adverse events together, the complication ratio per patient was more than double in cases receiving IO + tenotomy (1.18 versus 0.47).

**Table 2:** Complications of the surgical procedures performed on the lesser toes in both cohorts

	IO			IO + tenotomy			Fisher’s exact test significance	
	Events, *n*	Patients (%), *n* = 129	Procedures (%), *n* = 436	Events, *n*	Patients (%), *n* = 94	Procedures (%), *n* = 287	Patients	Procedures
Intraoperative phalangeal fracture	19	11.4	3.4	23	24.5	8.0	0.082	0.050
Postsurgical phalangeal fracture	3	1.8	0.5	12	12.8	4.2	0.002**	0.002**
Displacement	14	8.4	2.5	20	21.3	6.9	0.038*	0.003**
Delayed union	11	6.6	1.9	24	25.5	8.4	0.000‡	0.000‡
Hypertrophic callus	12	7.2	2.1	23	24.5	8.0	0.003**	0.002**
Non-union	0	–	–	1	1.1	0.3	0.421	0.397
Lack of correction	2	1.0	0.3	8	8.5	2.8	0.019*	0.017*

*IO* incomplete osteotomy

*p < 0.05; **p < 0.01; ^‡^*p* < 0.001

Cases operated on by IO + tenotomy showed a high rate of complications such as delayed union of the osteotomy (25.5% versus 6.6%; *p* < 0.01), hypertrophic callus (24.5% versus 7.2; *p* < 0.001), postoperative phalangeal fracture at the osteotomy site (12.8% versus 1.8%; *p* < 0.01), displacement (21.3% versus 8.4%; *p* < 0.05), and lack of correction (8.5% versus 1.0%; *p* < 0.05). The only case of non-union occurred in the IO + tenotomy cohort. No statistically significant differences were found between the two cohorts as regards the intraoperative breakage of the cortical wall although was most frequent in the IO + tenotomy cohort. However, within the first 30 days after surgery, 3 phalanges (2.3%) developed breakage in the group without tenotomy and 12 in the group with tenotomy (*p* < 0.05).

**Fig. 6:**
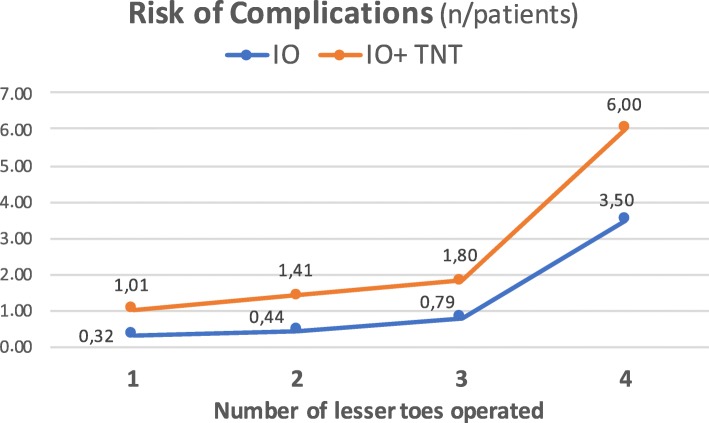
Risk of complications per patient in relation to the number of operated lesser toes

**Fig. 7:**
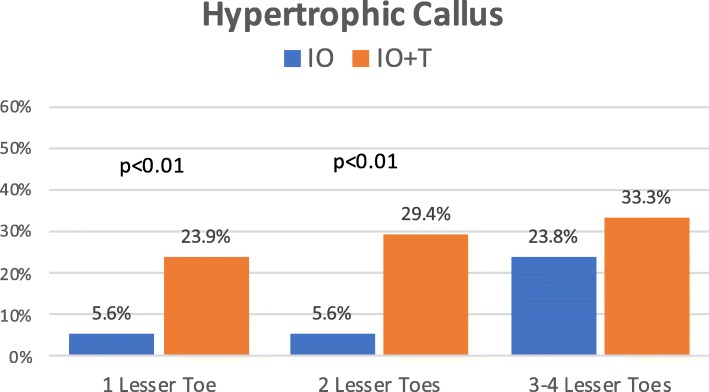
Occurrence of hypertrophic callus at the site of osteotomies in relation to the number of operated lesser toes (IO: complete osteotomy; T: tenotomy)

**Fig. 8:**
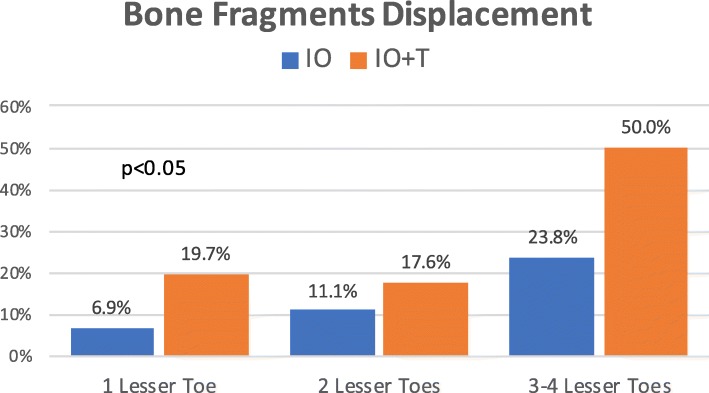
Occurrence of displacement of the bone fragments at the site of osteotomies in relation to the number of operated lesser toes (IO: complete osteotomy; T: tenotomy)

**Fig. 9.**
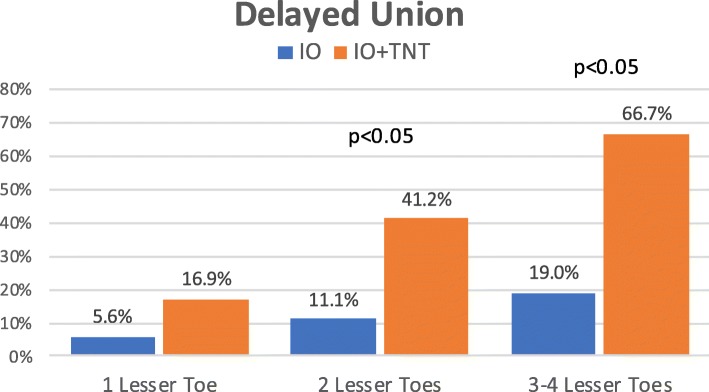
Occurrence of delayed union at the site of osteotomies in relation to the number of operated lesser toes (IO: complete osteotomy; T: tenotomy)

Figure 6 shows the risk of complications per patient in relation to the number of operated lesser toes. Differences between both cohorts were statistically significant at all stages, being complications more frequent in the tenotomy group. In each group, the percentage of osteotomies with displacement of bone fragments, delayed union, or development of a hypertrophic callus increased as the number of operated lesser toes increased (Figs. 7, 8, and 9). The increase is especially relevant when three or four lesser toes are operated.

Cases operated on without tenotomy showed a shorter time to complete recovery for daily life activities (37.4 ± 2.3 versus 43.0 ± 1.7 days; Mann Whitney test, *Z* = 11.813, *p* < 0.001).

## Discussion

To date, there is no consensus regarding the most appropriate surgical technique for LTD. Most likely, one of the reasons could be the wide variation in definitions of the deformities and indications for treatment. In a Dutch experience, a high proportion of orthopedic departments around the country had any protocol or agreement for the treatment of LTD [1]. This fact explained the broad spectrum in indications and performed interventions not only among the different hospitals but also within the departments.

Based on the latest evidence, several strategies for management of lesser toe deformities suggest different algorithms depending on the type of deformity [4]. These algorithms exclusively contemplate toe shortening procedures such as resection arthroplasty or arthrodesis, but never phalangeal osteotomies. For flexible mallet toes caused by a flexor digitus longus tight tendon, percutaneous flexor tenotomy is advocated to correct the deformity [3, 9].

The series include only cases with reducible hammer or claw toes, that is, flexible deformity of the proximal interphalangeal joint. Reducible hammer and claw toes were not considered clinically different and therefore were not analyzed separately within groups. Lesser toe deformities affecting the stiffness of the distal interphalangeal joint (rigid mallet toes) were excluded from the study because they commonly require resection arthroplasty or arthrodesis and cannot be treated by incomplete phalangeal osteotomies.

The treatments of lesser toes deformities by MIS are mostly based on soft tissue release, such as joint capsulotomies and tenotomies. The procedures on the bone depend on the residual deformities, the reducibility, and the toe length [11, 19, 20]. Some authors use complete osteotomies for the correction of digital deformities [21, 22], others perform unicortical osteotomies in certain pathologies [16, 23], but all of them follow the same protocol starting correction with soft tissue techniques.

Our proposal to treat LTD using IO techniques reverses the order of execution performing first the osteotomies in the bone segments, from proximal to distal. The procedures on soft tissues are finally carried out if the deformity requires additional actions in case of residual deformities. Recently, the European Federation of National Associations of Orthopaedics and Traumatology (EFORT) also suggests the addressing of proximal deformities prior to distal deformities [4, 9]. According to our practice, once the IOs are performed, the degree of correction and equilibrium achieved between the intrinsic and extrinsic muscle groups is intra-surgically assessed, then deciding whether or not to perform the flexor and/or extensor tenotomies.

The results obtained with the application of IO techniques (723 osteotomies in 556 toes) show a highly relevant functional improvement according to AOFAS scores. The current study addresses for the first time the clinical benefit of partial or incomplete phalangeal osteotomies by a commonly accepted measurement scale. AOFAS scores at 1-year follow-up show the clinical efficacy of IO technique using MIS for LTD. Outcomes are comparable to other studies performing digital arthrodesis and arthroplasty with open techniques [6–10, 16]. Although the AOFAS subscale focusing interphalangeal deformities of the lesser toes was only considered in this study, the results should be interpreted with caution. Some concerns have been raised with regard to AOFAS validity and reliability, but many clinicians continue to administer the AOFAS survey to patients. In fact, AOFAS scales continue to be some of the most widely used instruments in clinical studies, and they remain in use at a substantially higher rate than other scales that have been validated [23]. A relevant finding in our study is that the preoperative impairment of the AOFAS scores was dependent of the number of toes requiring surgical correction. Preoperative AOFAS scores decreased as the number of associated LTD increased. The clinical significance of these findings deserves further studies.

Most notably, the current study shows that the addition of flexor/extensor tenotomies to IO is also associated with good functional results, but worse than that found in cases without tenotomy. This finding is also confirmed by the earlier surgical discharge or the start of daily life activities in cases operated on by IO without tenotomies. Furthermore, the rate of adverse events was significantly higher when tenotomies were added to IO. Interestingly, in accordance with other MIS studies, no infection was found in the current series [24].

A commonly performed soft tissue MIS procedure in the lesser toes is a percutaneous flexor tenotomy. Debarge et al. [25] performed a percutaneous flexor tenotomy on 50 patients with clawing of the lesser toes secondary to shortening metatarsal scarf osteotomy. They found a 10% recurrence rate, and 4% of the patients developed complex regional pain syndrome. In cases with the same forefoot surgery but without flexor tenotomy, the rate of a toe grasping defect was significantly lower than in cases undergoing flexor tenotomy (4.3% versus 10%)

In the current series, the addition of a flexor/extensor tenotomy triggers some of the adverse events, particularly the percentage of delayed union, hypertrophic callus, bony fragments displacement, postsurgical phalangeal fracture, and final malunion. Previous studies indicate that some of these adverse events can influence outcome after lesser toe surgery [26]. On the contrary, most of the Dutch orthopedic surgeons (65%) stated that, when attempting interphalangeal arthrodesis, malunion and pseudarthrosis do not influence postoperative outcome [1]. Other authors showed that fusion was not required for a successful result following arthrodesis in cases with hammer toe [27].

In the present study, 57.8% of the operated toes by IO did not require additional tenotomies. This fact indicates that the alignment of the bone segments obtained with the osteotomies was able to reestablish the harmonic balance between the agonist and antagonist muscle groups, responsible for the digital mechanics, without the need to act on tendons. The new distribution of the force vectors on the bone surfaces could create an adequate compression and stability of the bone fragments. The design of the osteotomies preserving part of the cortical wall could help to reduce complications [28], providing a satisfactory functional recovery with a shorten postoperative period. The principal limitation of MIS to achieve satisfactory results is the way to perform and monitor the postoperative dressings that maintain toe alignment.

Diabetes mellitus, rheumatic diseases, and smoking seem to predispose to higher complication rates [26, 28]. As in the Gilheany et al. series [24], comorbidities did not translate to increased complication rates in the current cohorts. Similarly, none of our patients required an intraoperative conversion to open repair. Finally, the inclusion of patients of advanced age (over the age of 75) did not negatively influence both complication rate and outcome.

The stability provided by the cortical continuity when performing IO and the appropriate external bandages, responsible for closing the inter-fragmentary gap, maintains the contact between the bony surfaces. As IO preserves the articular surfaces, they are particularly indicated in reducible or semi-reducible digital deformities. In cases with joint dislocation or a deformity that requires shortening of the digit, IO combined with other MIS techniques such as complete osteotomies, toe arthroplasty, or arthrodesis would be desirable.

## Conclusion

Despite the limitations inherent to the variety of LTD, this study shows the clinical efficacy of the partial or incomplete osteotomies performed by MIS starting from proximal to distal phalanx in cases with reducible deformity of the proximal interphalangeal joint. Combined to the HAV correction, IO provides relevant functional improvement according to AOFAS functional scores. IO provides a satisfactory correction of LTD with functional and biomechanical restoration, and a low percentage of complications. These percutaneous techniques should be considered as an alternative to the conventional surgery of lesser toe deformities. Notably, the addition of flexor/extensor tenotomies to IO enhances the occurrence of adverse events particularly delayed union, hypertrophic callus, and postoperative phalangeal fracture. The avoidance of tenotomies supposes a lesser surgical aggression and a lower physiological response to the surgical trauma, achieving an earlier functional recovery. Further studies investigating the effectiveness of IO techniques are needed particularly focusing on long-term outcomes, as well developing treatment algorithms to guide clinical decision making.

## Data Availability

The datasets used and/or analyzed during the current study are available from the corresponding author on reasonable request.

## Abbreviations

AOFAS: American Orthopaedic Foot and Ankle Society
HVA: Hallux abductus valgus
IO: Incomplete osteotomy
LTD: Lesser toe deformities
MIS: Minimally invasive surgery
